# Effectiveness of Different Front-of-Pack Nutrition Labels among Italian Consumers: Results from an Online Randomized Controlled Trial

**DOI:** 10.3390/nu12082307

**Published:** 2020-07-31

**Authors:** Morgane Fialon, Manon Egnell, Zenobia Talati, Pilar Galan, Louise Dréano-Trécant, Mathilde Touvier, Simone Pettigrew, Serge Hercberg, Chantal Julia

**Affiliations:** 1 Sorbonne Paris Nord University, Inserm U1153, Inrae U1125, Cnam, Nutritional Epidemiology Research Team (EREN), Epidemiology and Statistics Research Center, University of Paris (CRESS), 93000 Bobigny, France; m.egnell@eren.smbh.univ-paris13.fr (M.E.); p.galan@uren.smbh.univ-paris13.fr (P.G.); louise.dreanotrecant@gmail.com (L.D.-T.); m.touvier@eren.smbh.univ-paris13.fr (M.T.); s.hercberg@eren.smbh.univ-paris13.fr (S.H.); julia@eren.smbh.univ-paris13.fr (C.J.); 2 School of Psychology, Curtin University, Kent St, Bentley, WA 6102, Australia; zenobia.talati@curtin.edu.au; 3 The George Institute for Global Health, Newtown, Sydney 2042, Australia; SPettigrew@georgeinstitute.org.au; 4 Public Health department, Avicenne Hospital, Assistance Publique des Hôpitaux de Paris (AP-HP), 93000 Bobigny, France

**Keywords:** nutritional labeling, food choices, comprehension, front-of-pack nutrition label, Italian consumers, Nutri-Score

## Abstract

In Italy, discussions are currently ongoing to implement a front-of-pack nutrition label (FoPL) while a growing number of European countries are adopting the Nutri-Score. The effectiveness of the Nutri-Score among Italian consumers requires further investigation. This study compared five FoPLs among Italian participants (Health Star Rating system, multiple traffic lights, Nutri-Score, reference intakes, warning symbol) in terms of food choices and understanding of the labels by consumers. In 2019, 1032 Italian consumers completed an online survey in which they were asked to select one product they would likely purchase from a set of three foods with different nutrient profiles and then classify the products within the set according to their nutritional quality, first with no label and then with one of the five FoPLs on the pack. While no significant difference across labels was observed for food choices, the Nutri-Score demonstrated the highest overall performance in helping consumers to correctly rank the products according to their nutritional quality compared to the reference intakes (OR = 2.18 (1.50–3.17), *p*-value < 0.0001). Our results provide new insights on the effectiveness of the Nutri-Score, which would be a relevant tool to inform Italian consumers on the nutritional quality of food products.

## 1. Introduction

Since recent decades, non-communicable diseases (NCDs) have become a major concern in Europe, accounting for about 77% of the burden of disease and 88% of death globally in 2017 [1]. In Italy, despite an increase in life expectancy since 2000, cardiovascular diseases and cancers remain the two leading causes of death, representing approximately two-thirds of all deaths in 2014 [2]. In parallel, the overweight and obesity rate among children in Italy is one of the highest across Europe, with nearly one-third of children suffering from overweight or obesity in 2016 [3]. Among the multiple contributing factors, nutrition is a common determinant of NCDs [4], and as reported by the Organisation for Economic Co-operation and Development, dietary risks accounted for about 16% of deaths in Italy in 2017 [5]. Nevertheless, dietary behavior is considered as a modifiable factor, providing an important opportunity for the prevention of NCDs in the long term [4].

Public health strategies have been proposed by international authorities, especially the World Health Organization, to improve dietary behaviors and prevent obesity and nutrition-related diseases. In recent years, the implementation of front-of-pack nutrition labels (FoPLs) has been of major interest to encourage healthier food choices [6,7,8] and have enticed manufacturers to improve the nutritional quality of the foods they offer [9,10]. Currently, given the European regulation, multiple schemes co-exist in the region [11], including nutrient-specific formats (e.g., multiple traffic lights (MTL) in the United Kingdom [12] and the reference intakes (RIs) implemented by several food manufacturers in Europe [13]), endorsement schemes (e.g., the Green Keyhole in Scandinavian countries [14]) or summary graded indicators (e.g., the Nutri-Score implemented in France, Belgium, Spain, the Netherlands, Germany, Luxembourg and Switzerland [15]). Recently, the European Commission “Farm to Fork” Strategy stipulated that a harmonized European FoPL is expected to be adopted in 2022 [16]. In Italy, politicians, as well as manufacturer and agricultural associations took a stance against the Nutri-Score, while this FoPL is strongly supported by several European consumer associations (including some Italian associations) and is being adopted by a growing number of European countries [17]. Recently, as an alternative to the Nutri-Score, the Italian Ministries of Health, Agriculture and Economic Development officially presented a new FoPL known as the NutrInform Battery, displaying the percentages of energy, fats, saturated fats, sugars and salt per portion, in relation to reference daily intakes: a format similar to the RIs label [18]. Although Italian stakeholders have raised criticisms against the Nutri-Score, to date no scientific comparative study has been published in Italy to test its effectiveness among consumers, in comparison to various FoPLs, such as the RIs, of which the NutrInform Battery is a variant. Using the method developed in the Front-Of-Pack International Comparative Experimental (FOP-ICE) study [19,20,21], the present paper investigates Italian consumers’ objective understanding of the Nutri-Score and four other FoPLs (Health Star Rating system (HSR), MTL, Nutri-Score, RIs, warning symbol), as well as the effect of these FoPLs on their food choices.

## 2. Materials and Methods

### 2.1. Population Study

In total, 1032 Italian adults were recruited through a web panel provider (Pureprofile), applying quotas for sex (50% of women), age (one third in each of the following categories: 18–30 years, 31–50 years, over 51 years) and monthly household income (one third in each of the following categories: low, medium and high). Income brackets were calculated by estimating the median household income within Italy (from national statistical databases) and creating a bracket of 33% around this figure. This represented the “medium” income band. Anything below or above this figure was considered as low- or high-income, respectively. Various methods, including online and mass media advertising and word-of mouth referrals, were used by the web panel provider to recruit panel members. Panel members were invited to complete an online survey in Italian. At the beginning of the survey, participants were asked to provide information on sex, age, monthly household income, educational level, involvement in grocery shopping, self-estimated diet quality (on a four-point scale between “I eat a very unhealthy diet” and “I eat a very healthy diet”) and nutrition knowledge (on a four-point scale between “I do not know anything about nutrition” and “I am very knowledgeable about nutrition”). The protocol of the study (same as the FOP-ICE study) was approved by the Institutional Review Board of the French Institute for Health and Medical Research (IRB Inserm n°17–404 bis) and the Curtin University Human Research Ethics Committee in Australia (approval reference: HRE2017–0760). Participants were invited to provide their electronic consent during the online survey.

### 2.2. Front-of-pack Nutrition Labels

Five FoPLs with different graphical designs were tested in the present study (Figure S1 [19,22]). Three nutrient-specific schemes were included: (i) the RIs, which provide only numerical information on energy and the content of fats, saturated fats, sugars and salt, including the contribution to the daily reference intakes [13]; (ii) MTL, which provides the same numerical information but with an associated color (red for high contents, orange for medium and green for low contents) [12]; and (iii) the warning symbol, that is applied to foods when the content in energy, saturated fats, sugars or sodium exceeds a specific level [23]. Two summary formats were also tested: (iv) the Nutri-Score, a graded and color-coded scheme characterizing the overall nutritional quality of products, from “A” in dark green for better nutritional quality to “E” in dark orange for lower nutritional quality [15]; and (v) the HSR, a hybrid format that provides information on the nutritional content as in the RIs, as well as a summary graded indicator of the overall nutritional quality of the food, using stars (from 0.5 to 5 stars) [24].

### 2.3. Design and Stimuli

Participants were exposed to three food categories, commonly available in Italian supermarkets and representing wide variability in nutritional quality: pizzas, cakes and breakfast cereals. In each food category, a set of three products with distinct nutrient profiles was created (higher, medium and lower nutritional quality), allowing a ranking of products according to their nutritional quality, which was similar across the FoPLs. To avoid potential bias in product evaluation (e.g., resulting from familiarity, habit), mock packages featuring a fictional brand (“Stofer”) were developed. When the FoPLs were applied on the mock packages in the second part of the study, they were affixed in the same place on each food and covered the same area on the package. To avoid unduly influencing participants’ evaluation of the food products, no other nutritional information or quality indicators were provided. The pizza stimuli are displayed in Figure S1 as an example [19]. All other stimuli have been described elsewhere [19]. In addition, at the beginning of the survey, participants were asked to declare the purchasing frequency of the tested food categories on a five-point scale (“Always”, “Often”, “Sometimes”, “Rarely” and “Never”). The participants who responded “Never” to at least two of the three food categories were excluded from the analyses to ensure that responses reflected real-world behaviors.

### 2.4. Procedure

Following the sociodemographic, lifestyle and nutrition-related questions at the beginning of the survey, participants were first asked to complete choice and understanding control tasks on the three food categories, without any label. For the three food categories, participants were first asked to select which of the three products they would buy, with an “I wouldn’t buy any of these products” option available, without any label on packages. Then, they were invited to rank the set of three products according to their nutritional quality (1—highest nutritional quality, 2—medium nutritional quality and 3—lowest nutritional quality), with an “I don’t know” option available, without any label as well. The phrasing of the task used relative terms on nutritional quality (highest/medium/lowest) in order to prevent participants from making assumptions about the absolute nutritional quality of the products. Choice and ranking tasks were completed with a randomized order of presentation of the food categories and products within the sets between respondents. Then, all participants were randomized to one of the five FoPL groups, with a balanced repartition between the five groups (i.e., approximately 200 participants per group). After the randomization, they were asked to complete the same choice and ranking tasks, but this time with an FoPL affixed on the mock packages, depending on the randomization arm. If a participant reported never purchasing products from a particular food category, his/her responses to the corresponding choice and ranking task were excluded. At the end of the questionnaire, participants were also invited to declare if they recalled having seen the FoPL they were exposed to during the online survey. Given the nature of the intervention (i.e., a nutrition label affixed on the front of food packages), participants were not blinded to the intervention, but they were blinded to the study objective. At the beginning of the survey, participants were only aware that the research project aimed to investigate factors affecting food choices; the presence of an FoPL during the second part of the study was never mentioned in order to not influence participants’ behavior, and no information on the different schemes was provided.

### 2.5. Outcome and Statistical Analysis

#### 2.5.1. Food Choice

For each food category, between 1 and 3 points were attributed to the choice tasks in both FoPL and no label conditions, with +1 point when the participant selected the lowest nutritional quality product, +2 for the intermediate nutritional quality product and +3 points for the highest nutritional quality product. No point was allocated when participants selected the “I wouldn’t buy any of these products” option, and the response was considered as missing. A choice score was then calculated for each food category using the difference in points between the FoPL and no label conditions, resulting in a discrete score ranging from −2 to +2 points. Finally, a global choice score was computed by summing the score of each category, ranging between −6 points (reflecting a deterioration of the nutritional quality of food choices) and +6 points (reflecting an improvement of food choices) for each participant. The percentage of participants who deteriorated or improved their choices between the no label and FoPL conditions was calculated for each FoPL group and food category. Associations between choice score and FoPL type were assessed using a multivariable ordinal logistic regression model, adjusted for sex, age, monthly household income, educational level, involvement in grocery shopping and self-estimated diet quality and nutrition knowledge. The models were performed on data from participants who selected a product in both no label and FoPL conditions. Several sensitivity analyses were performed to test the robustness of our results: (1) by conducting univariable models without any adjustment for individual characteristics; (2) by considering the choice outcome as a binary variable (i.e., choice score over 0 or not; (3) by comparing the choice scores between the five FoPLs conditions with the no label response as covariables; and (4) with an additional adjustment for food category purchasing frequency in models by category.

#### 2.5.2. Objective Understanding

Objective understanding of the FoPLs by consumers was measured by the ability of participants to correctly rank the products within each set according to nutritional quality. The ranking was considered correct when the three products within the set were correctly ranked, leading to a +1 point score for the category, while −1 point was allocated when the products were ranked out of order. Zero points were allocated when the participant selected the “I don’t know” answer. Thus, for each food category, a ranking score was calculated using the difference in points between the FoPL and no label conditions, ranging from −2 to +2 points, and leading to a global ranking score between −6 points (reflecting a deterioration of the participant ability to correctly rank products) and +6 points (reflecting an improvement) for the three food categories combined. The percentage of correct answers was computed by FoPL and food category. The association between FoPL type and the change in ability to correctly rank products according to nutritional quality was evaluated by a multivariable ordinal logistic regression model, adjusted for the same covariates as choice analyses.

The reference for the FoPL variable was the RIs in all choice and understanding models. Interactions between covariates and FoPLs were tested and stratified models were computed when the *p*-value of the interaction term was below 0.10. All analyses in the present study were conducted on the SAS statistical software (SAS Institute Inc., Cary, NC, USA, 2013); statistical tests were two-sided and a *p*-value below 0.05 was considered statistically significant. Several sensitivity analyses were performed to test the robustness of our results: (1) by conducting univariable models without any adjustment for individual characteristics; (2) by comparing the understanding scores between the five FoPLs conditions with the no label response as covariables; (3) with an additional adjustment for food category purchasing frequency in models by category; and (4) with an additional adjustment for the response to “Did you see this FoPL during the survey?

## 3. Results

### 3.1. Description of Individual Characteristics

Sociodemographic, lifestyle and nutrition-related characteristics of the study population are presented in Table 1. The sample included 1032 Italian participants, of whom 50% were women. Individuals over 50 years old represented 33% of the sample, 25% had a primary or secondary educational level and 34% had a low level of income. Additionally, 74% of participants declared they were responsible for grocery shopping, 10% having a very or mostly unhealthy self-estimated diet, and 13% declared having no or little knowledge about nutrition. A total of 63% of participants declared that they did recall having seen the label during the survey. Results varied across the schemes, but the HSR and the warning symbol were the two FoPLs with the lowest percentages of participants recalling having seen them. The description of the main sociodemographic characteristics of the sub-samples by FoPL group is displayed in Table S1. Overall, the distribution of individual characteristics was balanced between the five groups.

### 3.2. Food Choices

**Figure 1:**
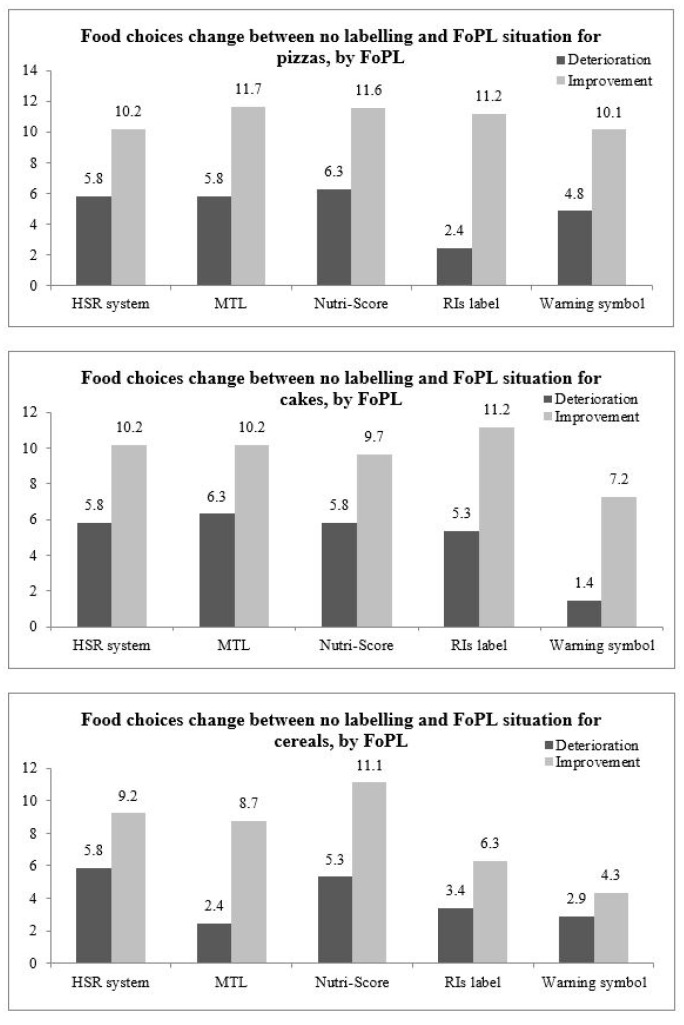
Percentage of participants having deteriorated or improved their food choices between the two choice tasks, by food category, in Italy.

Most of the participants did not change their food choices between the no label and FoPL conditions (between 59.4% and 77.1% depending on the label and the food category) or did not select any product in one or both of the labeling conditions (between 11.2% and 26.1%, depending on the label type and the food category). Between 2.4% and 11.7% of the participants, depending on the food category, changed their choice between the no label and FoPL conditions (Figure 1). The percentage of participants who improved their choices (between 4.3% and 11.7%, depending on the label and the food category) was higher than those having deteriorated their choices (between 1.4% and 6.3%, depending on the label and the food category). Globally, the five FoPLs demonstrated similar performance regarding the improvement and deterioration of the nutritional quality of food choices. Nevertheless, the relative performance of the various FoPLs varied across food categories.

The results of the ordinal logistic regression models are presented in Table 2. No significant association was found between any FoPLs and the change in nutritional quality of food choices compared to the RIs. All sensitivity analyses on food choices showed similar results compared to the main analyses (Tables S2–S5).

### 3.3. Objective Understanding

**Figure 2:**
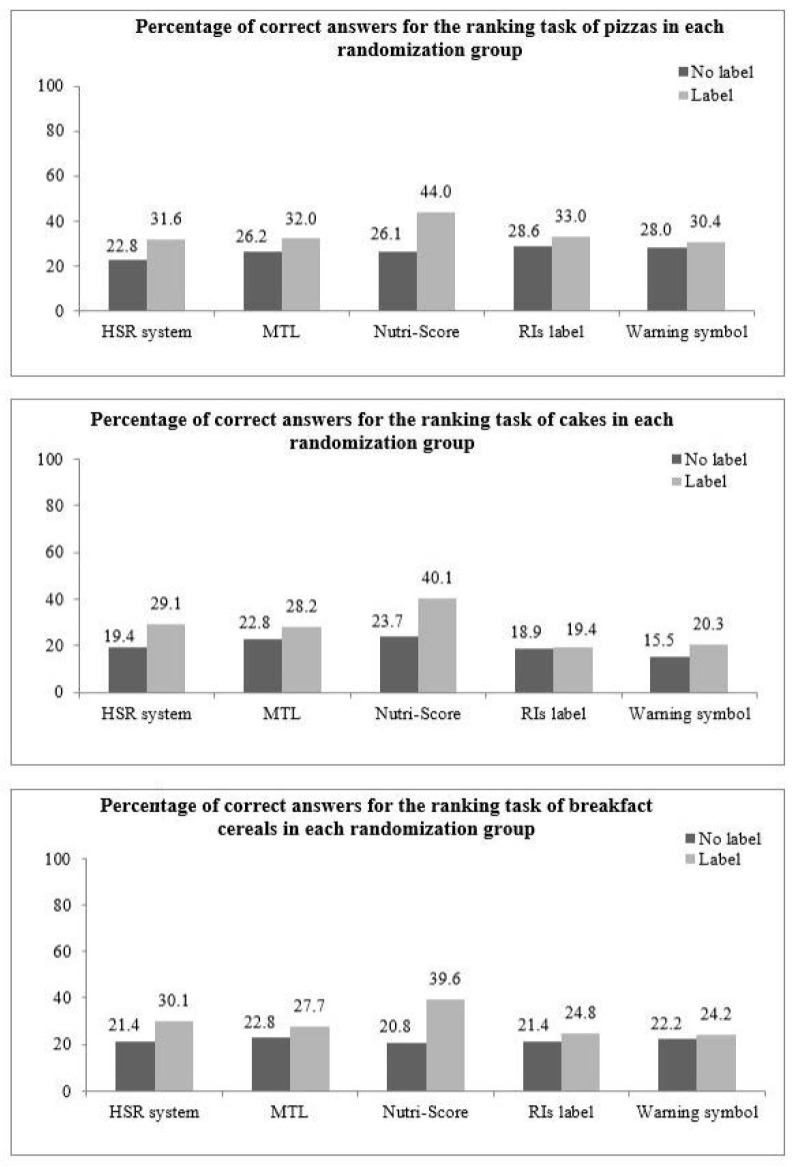
Percentage of correct answers with the change compared to no label, by FoPL and food category, in Italy.

The percentages of correct answers in the no label and FoPL conditions by FoPL type and food category are shown in Figure 2. The Nutri-Score was the FoPL demonstrating the highest increase in the percentage of correct answers between the no label and FoPL conditions in the three food categories (+17.9 percentage points for pizzas, +16.4 percentage points for cakes, +18.8 percentage points for breakfast cereals). The HSR showed the second-best performance across the three food categories (+8.8 percentage points for pizzas, +9.7 percentage points for cakes and +8.7 percentage points for breakfast cereals), followed by the MTL. In contrast, the RIs and the warning symbol were the two FoPLs inducing the smallest improvements in participants’ ability to correctly rank products according to their nutritional quality. A binomial test was performed to verify if the percentage of correct answers in the no label condition was statistically different from random guessing (16.67%) and a significant difference was observed (*p*-value < 0.0001).

Results of associations between FoPLs and participants’ ability to correctly rank products are displayed in Table 3. The Nutri-Score was the FoPL associated with the highest improvement in participants’ ability to correctly rank products compared to the RIs, both overall (OR = 2.18 (1.50–3.17), *p*-value < 0.0001) and for each of the three food categories (OR = 1.75 (1.11–2.76), *p*-value = 0.02 for pizzas; OR = 2.07 (1.34–3.20), *p*-value = 0.001 for cakes; OR = 2.56 (1.58–4.14), *p*-value = 0.0001 for breakfast cereals). The HSR had also a significant effect overall, but with lower amplitude effects (overall (OR = 1.59 (1.09–2.32), *p*-value = 0.02). The MTL and the warning symbol had no significant effect compared to the RIs. Compared to the other FoPLs, the Nutri-Score also performed significantly better than the MTL and the warning symbol overall only.

Interactions between FoPLs and individual characteristics (sex, age, educational level, monthly household income, responsibility for grocery shopping, self-estimated diet quality and nutrition knowledge) on the participants’ ability to correctly rank products were tested. No significant interactions were found. All other sensitivity analyses on the objective understanding of FoPLs by participants demonstrated similar results to the main analyses regarding the relative performance of the FoPLs (Tables S6–S9).

## 4. Discussion

While analyses did not find significant differences between FoPLs on change in food choices, significant differences were observed regarding the objective understanding outcome. Compared to the RIs, the Nutri-Score was the FoPL that produced the largest increase in participants’ ability to correctly rank the nutritional quality of products, followed by the HSR. These results are consistent with the findings based on the FOP-ICE study, where the Nutri-Score was observed as the best scheme to help participants identify the nutritional quality of products in different countries, including European countries: Belgium, Bulgaria, Denmark, France, Germany, Spain, Switzerland, the Netherlands and the United Kingdom [19,22,25,26]. Notably, similar results were observed in Spain, another Mediterranean country with a similar food context and dietary behaviors as Italy [19].

In this article, results for consumer understanding showed that interpretive FoPLs had greater potential than purely informative systems to improve the capacity of Italian consumers to correctly rank the nutritional quality of foods. This finding is consistent with the results of studies conducted in other countries [27,28,29,30,31,32,33,34,35], and especially European countries [19,36,37,38,39,40], with similar trends in FoPL outcomes identified in high- and middle-income countries. Moreover, it appears that summary indicators with a graded scale (i.e., Nutri-Score, HSR) are easier for consumers to understand as they showed the best results in the ranking tasks. This could be explained by the fact that their summary indicator requires a lower cognitive workload to process the information compared to nutrient-specific schemes [7,37,40]. However, between the two summary formats, the Nutri-Score outperformed the HSR in improving participants’ ability to correctly rank products according to nutritional quality compared to the RIs. This could be related to the use of color-coding from green to dark orange in the Nutri-Score format, increasing consumers’ attention [27,41] and helping individuals to process the information with the use of universal symbolic colors [42]. Moreover, from a biological perspective, dark orange and green are immediately discerned and discriminated by the human eye [43]. Thus, an FoPL combining both summary and color-coded features, such as the Nutri-Score, would be associated with a better objective understanding by consumers [15,19,39]. In contrast, non-interpretative FoPLs, such as the monochrome RIs label, similar to the Italian NutrInform Battery in its graphical format, have been observed to be poorly understood, especially among vulnerable populations [6,7,37,44,45,46]. This could be explained by the fact that nutrient-specific systems providing only numerical information could create confusion in nutritional terms and require a higher cognitive workload that can hinder their understanding and use in purchasing situations [6,7,37,44,45,46].

Regarding food choices, some surveys have shown that people reading back-of-pack information tend to have healthier eating intentions [47]. Nevertheless, this information is rarely used by consumers [48]. Studies investigating the effects of various FoPLs on the nutritional quality of consumers’ food choices or purchases have shown that interpretive schemes could guide consumers towards healthier foods. Indeed, several studies showed that interpretive systems including color-coding, graded scales or warning symbols, such as the Nutri-Score, the MTL, the HSR and warning symbol, appeared to be associated with healthier food choices [49,50,51,52,53,54,55,56,57,58], while the RIs schemes had little to no effect [52,53,54,57]. In our study, no significant differences were observed between the various FoPLs. This could be partly explained by the methodology used to test food choices in the present study. Indeed, it has been suggested that the effects of FoPLs on food choices depend on the choice task, such as the use of product sets rather than shopping carts, the type or number of products within the sets [59] or the assessed food categories [60]. When the effects of FoPLs on food choices were investigated in experimental or real supermarkets, the Nutri-Score demonstrated a positive effect on the nutritional quality of food purchases or purchasing intentions, while results were contrasted for other labels [15,53,61]. However, in the present study, the size of sets was determined by the ranking task; the ranking of the products’ nutritional quality had to be possible and similar with the five FoPLs, which would have been difficult with more than three products. Moreover, given that several dimensions were investigated in the same survey, the number of sets and products within the sets also had to be limited so that the questionnaire was not too long for participants to complete. Besides, effects of FoPLs on food choices may also have been underestimated for people who chose the best nutritional product in both the FoPL and no label conditions, as this was considered a “no change” outcome.

Strengths of our study are the inclusion of Italian consumers from various sociodemographic profiles, the investigation of two dimensions of FoPL effectiveness (i.e., objective understanding and food choices) and comparisons across multiple FoPL schemes using a randomized approach. A potential learning effect was also avoided by using randomization of the presentation order within choice sets and across food categories. It is important to note that comparative studies investigating the objective understanding of FoPLs provide scientific evidence on an important pre-requisite of FoPL use (i.e., consumers’ ability to correctly use the FoPL), and also permit assessment of relative performances of different schemes. Nevertheless, some limitations should be noted. First, Italian participants were recruited online using quotas, which would limit the generalizability of the findings and requires caution regarding the extrapolation of the results to the population as a whole. However, the recruitment aimed to include sub-groups of populations who are more difficult to access in research and for whom FoPL effectiveness can vary rather than obtain a representative sample. In addition, the quota sampling recruitment allowed performing cross-cultural comparisons of FoPL effectiveness between multiple countries within the FOP-ICE study. Second, as the participants were not provided with the meaning of the FoPL to which they were exposed and the objective of the study, it may have led to an underestimation of the labels’ effects, especially for unknown FoPLs—the RIs label implemented in Europe was the FoPL that participants were most likely to be familiar with. However, this bias should have impacted all FoPLs. Further, participants did not have access to the nutritional composition of the products used in the study. The absence of this element might have led to fewer correct responses in the no label condition in the understanding task compared to what would occur in real-life settings, resulting in overestimation of the FoPLs’ effects. However, the literature shows that back-of-pack information is rarely used by consumers [48]. This limitation also applied equally to all labels included in the study and should not have influenced the differences between schemes. Furthermore, the stimuli were developed to ensure a clear nutritional difference between the products using each FoPL. However, this methodological choice with sets of three products led to the exclusion of endorsement schemes from the test as the classification of products with these FoPLs is difficult to assess across more than two products at once (e.g., no discrimination would be possible between two products without any endorsement labels on their packages). Moreover, the study was conducted as an online experiment and not in a real shopping situation in which many additional factors such as prices are likely influence consumers’ choices. Future research is needed that includes price as an additional variable to assess the extent to which cost considerations may impact on FoPL effectiveness. Finally, the results could have been impacted by familiarity and purchasing habits for the food categories used in the study. Nevertheless, this bias was minimized by the use of fictional products and a fictional brand and the exclusion of the responses of participants who declared having never purchased one of the food categories.

## 5. Conclusions

The present findings provide new evidence on the effectiveness of various FoPLs among Italian consumers. All five FoPLs improved somewhat the nutritional quality of food choices compared to no label for some participants, with no significant differences across schemes. However, the Nutri-Score was the best format to help Italian consumers identify the nutritional quality of foods. On the contrary, the RIs label, the format of which is very close to the new FoPL format being proposed by the Italian government, the NutrInform Battery, was the least efficient scheme to assist Italian consumers in interpreting the nutritional quality of foods.

## Figures and Tables

**Table 1:** Description of the population sample from Italy (*N* = 1032 participants).

Variable	*N* (%)
Sex	
Men	515 (49.90)
Women	517 (50.10)
Age, years	
18–30	347 (33.62)
31–50	343 (33.24)
> 50	342 (33.14)
Educational level	
Primary education	16 (1.55)
Secondary education	240 (23.26)
Trade certificate	259 (25.10)
University, undergraduate degree	289 (28.00)
University postgraduate degree	228 (22.09)
Level of household income	
High	342 (33.14)
Medium	343 (33.24)
Low	347 (33.62)
Responsible for grocery shopping	
Yes	765 (74.13)
No	50 (4.84)
Share job equally	217 (21.03)
Self-estimated diet quality	
I eat a very unhealthy diet	1 (0.10)
I eat a mostly unhealthy diet	104 (10.08)
I eat a mostly healthy diet	787 (76.26)
I eat a very healthy diet	140 (13.57)
Self-estimated nutrition knowledge	
I do not know anything about nutrition	3 (0.29)
I am not very knowledgeable about nutrition	132 (12.79)
I am somewhat knowledgeable about nutrition	746 (72.29)
I am very knowledgeable about nutrition	151 (14.63)
Did you see the label during the survey?	
No	316 (30.62)
Unsure	68 (6.59)
Yes	648 (62.79)
Participants who recalled seeing the label they were exposed to	
Health Star Rating system	108 (52.4)
Multiple traffic lights	149 (72.3)
Nutri-Score	130 (62.8)
Reference intakes	162 (78.6)
Warning symbol	99 (47.8)

**Table 2:** Associations between the front-of-pack nutrition labels (FoPLs) and the change in nutritional quality of food choices.

Food Category	*N*	HSR		MTL		Nutri-Score		Warning Symbol	
		OR (95% CI)	*p*	OR (95% CI)	*p*	OR (95% CI)	*p*	OR (95% CI)	*p*
All categories	984	0.87 (0.57–1.32)	0.5	0.97 (0.64–1.48)	0.9	1.01 (0.67–1.54)	1.0	0.92 (0.60–1.41)	0.7
Pizzas	848	0.74 (0.43–1.27)	0.3	0.81 (0.48–1.38)	0.4	0.82 (0.48–1.39)	0.5	0.80 (0.46–1.39)	0.4
Cakes	817	0.86 (0.50–1.50)	0.6	0.86 (0.49–1.50)	0.6	0.91 (0.52–1.60)	0.7	1.01 (0.57–1.77)	1.0
Breakfast cereals	862	1.18 (0.64–2.17)	0.6	1.47 (0.80–2.68)	0.2	1.52 (0.83–2.77)	0.2	0.97 (0.52–1.80)	0.9

The reference of the multivariate ordinal logistic regression for the categorical variable “label” was the reference intakes. The multivariate model was adjusted for sex, age, educational level, level of household income, responsibility for grocery shopping and self-estimated diet quality and nutrition knowledge level. HSR: Health Star Rating system; MTL: multiple traffic lights; OR: odds ratio; CI: confidence interval; *p*: *p*-value. Bold values correspond to significant results (*p*-value ≤ 0.05).

**Table 3:** Associations between FoPLs and change in ability to correctly rank products between no label and labeling conditions.

Food Category	*N*	HSR		MTL		Nutri-Score		Warning Symbol	
		OR (95% CI)	*p*	OR (95% CI)	*p*	OR (95% CI)	*p*	OR (95% CI)	*p*
All categories	1032	1.59 (1.09–2.32)	**0.02**	1.01 (0.69–1.47)	1.0	2.18 (1.50–3.17)	**<0.0001**	1.02 (0.70–1.49)	0.9
Pizzas	1022	1.31 (0.83–2.08)	0.2	1.09 (0.68–1.73)	0.7	1.75 (1.11–2.76)	**0.02**	0.84 (0.52–1.33)	0.5
Cakes	1028	1.57 (1.01–2.43)	**0.04**	1.10 (0.71–1.71)	0.7	2.07 (1.34–3.20)	**0.001**	1.26 (0.81–1.96)	0.3
Breakfast cereals	963	1.77 (1.08–2.90)	**0.02**	1.02 (0.62–1.68)	0.9	2.56 (1.58–4.14)	**0.0001**	1.06 (0.65–1.74)	0.8

The reference of the multivariate ordinal logistic regression for the categorical variable “label” was the reference intakes. The multivariate model was adjusted for sex, age, educational level, level of income, responsibility for grocery shopping, self-estimated diet quality and self-estimated nutrition knowledge level. HSR: Health Star Rating system; MTL: multiple traffic lights; OR: odds ratio; CI: confidence interval; *p*: *p*-value. Bold values correspond to significant results (*p*-value ≤ 0.05).

## Supplementary Materials

The following are available online at https://www.mdpi.com/2072-6643/12/8/2307/s1, Figure S1: Example of the set of three pizzas with the corresponding labeling conditions; Table S1: Description of the main individual characteristics by FoPL group, N (%); Table S2: Associations between the FoPLs and the change in nutritional quality of food choices using univariable models; Table S3: Associations between the FoPLs and the change in nutritional quality of food choices, using a binary outcome; Table S4: Associations between the FoPLs and the nutritional quality of food choices in the labeling condition; Table S5: Associations between the FoPLs and the change in nutritional quality of food choices, taking into account the purchasing frequency of food categories; Table S6: Associations between FoPLs and change in ability to correctly rank products between no label and labeling conditions, using univariable models; Table S7: Associations between FoPLs and the ability to correctly rank products in the labeling condition; Table S8: Associations between FoPLs and change in ability to correctly rank products between no label and labeling conditions, taking into account the purchasing frequency of food categories; Table S9: Associations between FoPLs and change in ability to correctly rank products between no label and labeling conditions, taking into account whether the participants recalled seeing the FoPL during the survey.
