# Supplementary material for: Effectiveness of Different Front-of-Pack Nutrition Labels among Italian Consumers: Results from an Online Randomized Controlled Trial
Source: Nutrients. 2020 Jul 31;12(8):2307. doi: 10.3390/nu12082307 (PMC7468990; doi:10.3390/nu12082307)
Supplement: Supplementary file 1 [file nutrients-12-02307-s001.pdf]

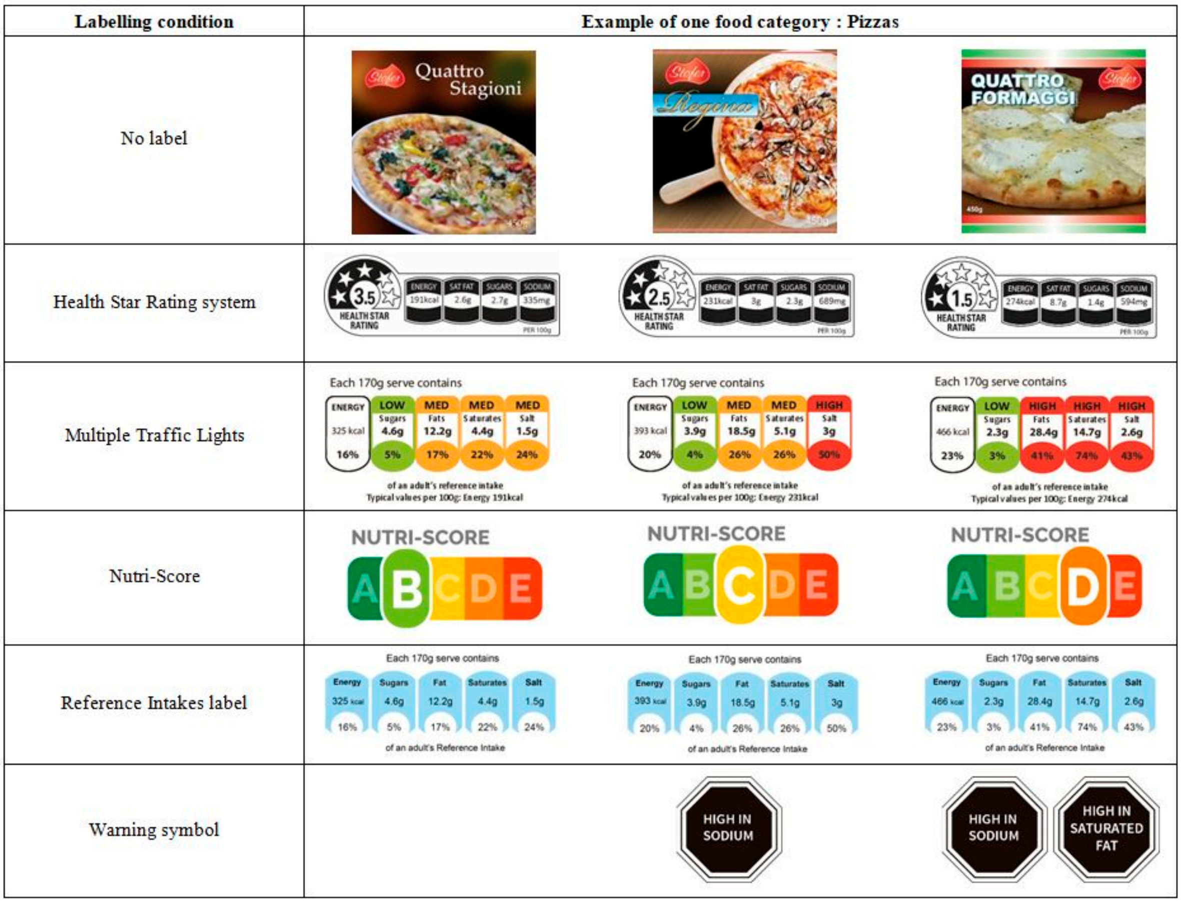

Figure 1. Example of the set of three pizzas with the corresponding labeling conditions.

Table S1. Description of the main individual characteristics by FoPL group, N (%).

Variable	HSR (*N* = 206)	MTL (*N* = 206)	Nutri–Score (*N* = 207)	Reference Intakes (*N* = 206)	Warning Symbol (*N* = 207)
Sex					
Men	107 (51.94)	95 (46.12)	102 (49.28)	111 (53.88)	100 (48.31)
Women	99 (48.06)	111 (53.88)	105 (50.72)	95 (46.12)	107 (51.69)
Age, Years					
18–30	71 (34.47)	70 (33.98)	66 (31.88)	72 (34.95)	68 (32.85)
31–50	65 (31.55)	68 (33.01)	70 (33.82)	75 (36.41)	65 (31.40)
> 50	70 (33.98)	68 (33.01)	71 (34.30)	59 (28.64)	74 (35.75)
Educational level					
Primary education	3 (1.46)	5 (2.43)	2 (0.97)	0 (0.00)	6 (2.90)
Secondary education	55 (26.70)	47 (22.82)	49 (23.67)	43 (20.87)	46 (22.22)
Trade certificate	47 (22.82)	57 (27.67)	52 (25.12)	48 (23.30)	55 (26.57)
University, undergraduate degree	46 (22.33)	44 (21.36)	44 (21.26)	50 (24.27)	44 (21.26)
University, Postgraduate degree	55 (26.70)	53 (25.73)	60 (28.99)	65 (31.55)	56 (27.05)
Level of household income					
High	71 (34.47)	67 (32.52)	67 (32.37)	61 (29.61)	76 (36.71)
Medium	66 (32.04)	62 (30.10)	70 (33.82)	78 (37.86)	71 (34.30)
Level	69 (33.50)	77 (37.38)	70 (33.82)	67 (32.52)	60 (28.99)

HSR: Health Star Rating system; MTL: multiple traffic lights.

Table S2. Associations between the FoPLs and the change in nutritional quality of food choices using univariable models.

Food category	*N*	HSR		MTL		Nutri–Score		Warning symbol	
		OR (95% CI)	*P*	OR (95% CI)	*P*	OR (95% CI)	*P*	OR (95% CI)	*P*
**All categories**	984	0.85 (0.56–1.29)	0.4	0.96 (0.63–1.45)	0.8	0.99 (0.65–1.49)	1.0	0.90 (0.59–1.37)	0.6
Pizzas	848	0.76 (0.45–1.29)	0.3	0.84 (0.50–1.41)	0.5	0.83 (0.49–1.40)	0.5	0.84 (0.49–1.44)	0.5
Cakes	817	0.92 (0.54–1.58)	0.8	0.91 (0.53–1.57)	0.7	0.93 (0.53–1.61)	0.8	1.05 (0.61–1.83)	0.9
Breakfast cereals	862	1.09 (0.59–2.00)	0.8	1.39 (0.77–2.51)	0.3	1.35 (0.75–2.44)	0.3	0.90 (0.49–1.66)	0.7

The reference of the ordinal logistic regression for the categorical variable "label" was the reference intakes. Models were not adjusted. HSR: Health Star Rating system; MTL: multiple traffic lights; OR: odds ratio; CI: confidence interval; *P*: *P*–value. Bold values correspond to significant results (*P*–value ≤ 0.05).

Table S3. Associations between the FoPLs and the change in nutritional quality of food choices, using a binary outcome.

Food Category	*N*	HSR		MTL		Nutri–Score		Warning Symbol	
		OR (95% CI)	*P*	OR (95% CI)	*P*	OR (95% CI)	*P*	OR (95% CI)	*P*
All categories	984	0.92 (0.48–1.74)	0.8	1.06 (0.57–1.98)	0.9	1.09 (0.58–2.04)	0.8	1.02 (0.53–1.96)	0.9
Pizzas	848	0.94 (0.49–1.79)	0.8	0.89 (0.47–1.71)	0.7	0.96 (0.50–1.85)	0.9	0.66 (0.33–1.34)	0.3
Cakes	817	1.82 (0.85–3.91)	0.1	1.67 (0.77–3.60)	0.2	2.17 (1.03–4.58)	0.04	0.81 (0.33–2.00)	0.7
Breakfast cereals	862	0.92 (0.48–1.74)	0.8	1.06 (0.57–1.98)	0.9	1.09 (0.58–2.04)	0.8	1.02 (0.53–1.96)	0.9

The reference of the ordinal logistic regression for the categorical variable "label" was the reference intakes. The multivariate model was adjusted for sex, age, educational level, level of income, responsibility for grocery shopping, self–estimated diet quality and self–estimated nutrition knowledge level. Choices was coded as a binary outcome (i.e., choice score > 0 or not). HSR: Health Star Rating system; MTL: multiple traffic lights; OR: odds ratio; CI: confidence interval; *P*: *P*–value. Bold values correspond to significant results (*P*–value ≤ 0.05).

Table S4. Associations between the FoPLs and the nutritional quality of food choices in the labeling condition.

Food Category	*N*	HSR		MTL		Nutri–Score		Warning Symbol	
		OR (95% CI)	*P*	OR (95% CI)	*P*	OR (95% CI)	*P*	OR (95% CI)	*P*
All categories	984	0.91 (0.63–1.32)	0.6	0.94 (0.65–1.35)	0.7	1.16 (0.80–1.67)	0.4	0.76 (0.52–1.10)	0.1
Pizzas	848	0.65 (0.39–1.06)	0.08	0.81 (0.49–1.33)	0.4	0.77 (0.47–1.26)	0.3	0.73 (0.44–1.22)	0.2
Cakes	817	0.87 (0.54–1.38)	0.6	0.94 (0.59–1.51)	0.8	1.01 (0.63–1.64)	1.0	1.00 (0.62–1.61)	1.0
Breakfast cereals	862	0.98 (0.56–1.73)	1.0	1.30 (0.73–2.32)	0.4	1.50 (0.83–2.70)	0.2	1.01 (0.55–1.86)	1.0

The reference of the ordinal logistic regression for the categorical variable "label" was the reference intakes. The multivariate model was adjusted for sex, age, educational level, level of income, responsibility for grocery shopping, self–estimated diet quality, self–estimated nutrition knowledge level and the choice score in the no label condition. HSR: Health Star Rating system; MTL: multiple traffic lights; OR: odds ratio; CI: confidence interval; *P*: *P*–value. Bold values correspond to significant results (*P*–value ≤ 0.05).

Table S5. Associations between the FoPLs and the change in nutritional quality of food choices, taking into account the purchasing frequency of food categories.

Food Category	*N*	HSR		MTL		Nutri–Score		Warning Symbol	
		OR (95% CI)	*P*	OR (95% CI)	*P*	OR (95% CI)	*P*	OR (95% CI)	*P*
Pizzas	848	0.75 (0.44–1.29)	0.3	0.80 (0.47–1.37)	0.4	0.82 (0.48–1.40)	0.5	0.78 (0.45–1.35)	0.4
Cakes	817	0.86 (0.50–1.49)	0.6	0.85 (0.49–1.48)	0.6	0.90 (0.52–1.59)	0.7	1.00 (0.57–1.76)	1.0
Breakfast cereals	862	1.16 (0.63–2.15)	0.6	1.45 (0.79–2.64)	0.2	1.50 (0.82–2.74)	0.2	0.96 (0.52–1.79)	0.9

The reference of the ordinal logistic regression for the categorical variable "label" was the reference intakes.

The multivariate model was adjusted for sex, age, educational level, level of income, responsibility for grocery shopping, self–estimated diet quality, self–estimated nutrition knowledge level and the purchasing frequency of the corresponding food category. HSR: Health Star Rating system; MTL: multiple traffic lights; OR: odds ratio; CI: confidence interval; *P*: *P*–value. Bold values correspond to significant results (*P*–value ≤ 0.05).

Table S6. Associations between FoPLs and change in ability to correctly rank products between no label and labeling conditions, using univariable models.

Food Category	*N*	HSR		MTL		Nutri–Score		Warning Symbol	
		OR (95% CI)	*P*	OR (95% CI)	*P*	OR (95% CI)	*P*	OR (95% CI)	*P*
All categories	1032	1.56 (1.07–2.26)	**0.02**	1.01 (0.7–1.47)	0.9	2.16 (1.49–3.13)	**< 0.0001**	1.03 (0.71–1.49)	0.9
Pizzas	1022	1.28 (0.81–2.02)	0.3	1.06 (0.67–1.68)	0.8	1.81 (1.16–2.83)	**0.01**	0.82 (0.52–1.31)	0.4
Cakes	1028	1.57 (1.02–2.42)	**0.04**	1.11 (0.72–1.72)	0.6	2.04 (1.33–3.13)	**0.001**	1.26 (0.81–1.94)	0.3
Breakfast cereals	963	1.77 (1.08–2.89)	**0.02**	1.04 (0.64–1.71)	0.9	2.60 (1.61–4.18)	**< 0.0001**	1.08 (0.66–1.76)	0.8

The reference of the ordinal logistic regression for the categorical variable "label" was the reference intakes. Models were not adjusted. HSR: Health Star Rating system; MTL: multiple traffic lights; OR: odds ratio; CI: confidence interval; *P*: *P*–value. Bold values correspond to significant results (*P*–value ≤ 0.05).

Table S7. Associations between FoPLs and the ability to correctly rank products in the labeling condition.

Food Category	*N*	HSR		MTL		Nutri–Score		Warning Symbol	
		OR (95% CI)	*P*	OR (95% CI)	*P*	OR (95% CI)	*P*	OR (95% CI)	*P*
All categories	1032	1.55 (1.08–2.22)	**0.02**	1.12 (0.78–1.61)	0.5	2.33 (1.63–3.35)	**< 0.0001**	1.07 (0.75–1.54)	0.7
Pizzas	1022	1.18 (0.76–1.82)	0.5	1.02 (0.66–1.58)	0.9	1.77 (1.15–2.72)	**0.01**	0.95 (0.61–1.47)	0.8
Cakes	1028	1.71 (1.12–2.62)	**0.01**	1.37 (0.89–2.10)	0.2	2.53 (1.66–3.86)	**< 0.0001**	1.28 (0.82–1.97)	0.3
Breakfast cereals	963	1.81 (1.12–2.92)	**0.01**	1.15 (0.71–1.85)	0.6	2.60 (1.63–4.13)	**< 0.0001**	1.15 (0.72–1.85)	0.6

The reference of the ordinal logistic regression for the categorical variable "label" was the reference intakes. The multivariate model was adjusted for sex, age, educational level, level of income, responsibility for grocery shopping, self–estimated diet quality, self–estimated nutrition knowledge level and the understanding score in the no label condition. HSR: Health Star Rating system; MTL: multiple traffic lights; OR: odds ratio; CI: confidence interval; *P*: *P*–value. Bold values correspond to significant results (*P*–value ≤ 0.05).

Table S8. Associations between FoPLs and change in ability to correctly rank products between no label and labeling conditions, taking into account the purchasing frequency of food categories.

Food Category	*N*	HSR		MTL		Nutri–Score		Warning Symbol	
		OR (95% CI)	*P*	OR (95% CI)	*P*	OR (95% CI)	*P*	OR (95% CI)	*P*
**Pizzas**	1022	1.31 (0.83–2.08)	0.2	1.08 (0.68–1.72)	0.7	1.74 (1.11–2.74)	**0.02**	0.83 (0.52–1.33)	0.4
Cakes	1028	1.59 (1.03–2.47)	**0.04**	1.07 (0.69–1.67)	0.8	2.03 (1.32–3.14)	**0.001**	1.27 (0.82–1.98)	0.3
Breakfast cereals	963	1.79 (1.09–2.94)	**0.02**	1.03 (0.62–1.69)	0.9	2.56 (1.58–4.16)	**0.0001**	1.06 (0.65–1.74)	0.8

The reference of the ordinal logistic regression for the categorical variable "label" was the reference intakes. The multivariate model was adjusted for sex, age, educational level, level of income, responsibility for grocery shopping, self–estimated diet quality, self–estimated nutrition knowledge level and the purchasing frequency of the corresponding food category. HSR: Health Star Rating system; MTL: multiple traffic lights; OR: odds ratio; CI: confidence interval; *P*: *P*–value. Bold values correspond to significant results (*P*–value ≤ 0.05).

Table S9. Associations between FoPLs and change in ability to correctly rank products between no label and labeling conditions, taking into account whether the participants recalled seeing the FoPL during the survey.

Food Category	*N*	HSR		MTL		Nutri–Score		Warning Symbol	
		OR (95% CI)	*P*	OR (95% CI)	*P*	OR (95% CI)	*P*	OR (95% CI)	*P*

**All categories**	1032	1.77 (1.2–2.59)	**0.004**	1.06 (0.72–1.54)	0.8	2.33 (1.6–3.4)	**< 0.0001**	1.19 (0.80–1.75)	0.4
Pizzas	1022	1.47 (0.92–2.34)	0.1	1.12 (0.71–1.79)	0.6	1.89 (1.2–2.99)	**0.006**	0.97 (0.6–1.57)	0.9
Cakes	1028	1.63 (1.04–2.54)	**0.03**	1.13 (0.73–1.76)	0.6	2.15 (1.39–3.33)	**0.0006**	1.37 (0.87–2.15)	0.2
Breakfast cereals	963	1.97 (1.19–3.26)	**0.008**	1.08 (0.66–1.78)	0.8	2.76 (1.7–4.49)	**< 0.0001**	1.26 (0.76–2.10)	0.4

The reference of the ordinal logistic regression for the categorical variable "label" was the reference intakes. The multivariate model was adjusted for sex, age, educational level, level of income, responsibility for grocery shopping, self–estimated diet quality, self–estimated nutrition knowledge level and the response to the question "Did you see this FoPL during the survey? HSR: Health Star Rating system; MTL: multiple traffic lights; OR: odds ratio; CI: confidence interval; *P*: *P*–value. Bold values correspond to significant results (*P*–value ≤ 0.05).
